# Preimplantation factor modulates trophoblastic invasion throughout the decidualization of human endometrial stromal cells

**DOI:** 10.1186/s12958-021-00774-5

**Published:** 2021-06-27

**Authors:** Esther Dos Santos, Hadia Moindjie, Valérie Sérazin, Lucie Arnould, Yoann Rodriguez, Khadija Fathallah, Eytan R. Barnea, François Vialard, Marie-Noëlle Dieudonné

**Affiliations:** 1 Université Paris-Saclay, UVSQ, INRAE, BREED, F-78350 Jouy-en-Josas, France; 2 Ecole Nationale Vétérinaire d’Alfort, BREED, F-94700 Maisons-Alfort, France; 3 Service de Biologie Médicale, Centre Hospitalier de Poissy-Saint Germain, F-78300 Poissy, France; 4 INSERM- UMR 981 Biomarqueurs prédictifs et nouvelles stratégies thérapeutiques en oncologie. Bâtiment Médecine Moléculaire (B2M), 114 Rue Edouard Vaillant, 94800 Villejuif, France; 5 Département de Biologie de la Reproduction, Cytogénétique, Gynécologie et Obstétrique, Centre Hospitalier de Poissy-Saint Germain, F-78300 Poissy, France; 6 Society for the Investigation of Early Pregnancy, Cherry Hill, NJ USA; 7 BioIncept, LLC, Cherry Hill, NJ USA; 8 UMR 1198 BREED-RHuMA, Université de Versailles-Saint Quentin en Yvelines - Université Paris Saclay, UFR des Sciences de la Santé Simone Veil, 2 Avenue de la Source de la Bièvre, F-78180 Montigny-le-Bretonneux, France

**Keywords:** Preimplantation factor (PIF), Human endometrium, Decidualization, Embryo implantation, Trophoblastic invasion, Signal transduction

## Abstract

**Background:**

Successful human embryo implantation requires the differentiation of endometrial stromal cells (ESCs) into decidual cells during a process called decidualization. ESCs express specific markers of decidualization, including prolactin, insulin-like growth factor-binding protein-1 (IGFBP-1), and connexin-43. Decidual cells also control of trophoblast invasion by secreting various factors, such as matrix metalloproteinases (MMPs) and tissue inhibitors of metalloproteinases. Preimplantation factor (PIF) is a recently identified, embryo-derived peptide with activities at the fetal-maternal interface. It creates a favorable pro-inflammatory environment in human endometrium and directly controls placental development by increasing the human trophoblastic cells’ ability to invade the endometrium. We hypothesized that PIF’s effects on the endometrium counteract its pro-invasive effects.

**Methods:**

We tested sPIF effect on the expression of three decidualization markers by RT-qPCR and/or immunochemiluminescence assay. We examined sPIF effect on human ESC migration by performing an in vitro wound healing assay. We analyzed sPIF effect on endometrial control of human trophoblast invasion by performing a zymography and an invasion assay.

**Results:**

Firstly, we found that a synthetic analog of PIF (sPIF) significantly upregulates the mRNA expression of IGFBP-1 and connexin-43, and prolactin secretion in ESCs - suggesting a pro-differentiation effect. Secondly, we showed that the HTR-8/SVneo trophoblastic cell line’s invasive ability was low in the presence of conditioned media from ESCs cultured with sPIF. Thirdly, this PIF’s anti-invasive action was associated with a specifically decrease in MMP-9 activity.

**Conclusion:**

Taken as a whole, our results suggest that PIF accentuates the decidualization process and the production of endometrial factors that limit trophoblast invasion. By controlling both trophoblast and endometrial cells, PIF therefore appears to be a pivotal player in the human embryo implantation process.

## Background

Human embryo implantation is a multistep process that begins with the apposition of trophoblastic cells from a competent blastocyst against the maternal endometrium. This apposition occurs during the “implantation window” - a short period of uterine receptivity corresponding to the mid-secretory phase of the menstrual cycle [1]. After the blastocyst has attached to the endometrial epithelium, its extravillous trophoblasts (EVTs) penetrate into the endometrial stroma and acquire an invasive phenotype - resulting in the placenta anchoring in the endometrium [2].

During trophoblast invasion, EVTs secrete large amounts of matrix metalloproteinases (MMPs) [3]. The actions of MMP-2 and MMP-9 are crucial for trophoblast invasion [4]. Indeed, these gelatinases are able to degrade the major component of the endometrial extracellular matrix (ECM), i.e. collagen IV. The level of MMP activity is regulated by specific tissue inhibitors of metalloproteinases (TIMPs), which bind to and inactivate MMPs with a 1:1 stoichiometry [5]. Thus, correct regulation of the MMP/TIMP balance in EVTs is essential for successful embryo implantation [6]. Trophoblast invasion also requires adequate endometrial receptivity [7]. This is characterized by morphological, biochemical, and vascular changes, including (i) the appearance of epithelial pinopods, (ii) the modulated expression of cytokines, growth factors, and adhesion molecules (particularly the upregulation of αv- and β3-integrins and the disappearance of mucin-1 and mucin-12 in endometrial epithelial cells), and (iii) the decidualization of endometrial stromal cells (ESCs). Decidualization occurs in response to the ovarian hormones 17β-estradiol (E2) and progesterone (P4). It is accompanied by the secretion of prolactin and insulin-like growth factor-binding protein-1 (IGFBP-1), and the expression of the gap junction connexin-43 (CX-43) in human ESCs [8, 9]. Decidual ECM remodeling events have also been observed. These processes are associated with MMP-2,-9 secretion and the modulation of *TIMP-1,-2* gene expression in human ESCs [10–12]. This type of ECM remodeling enhances ESC motility and thus facilitates trophoblastic infiltration into the endometrial stroma [13]. Decidual ECM remodeling is a complex process that is tightly controlled by intrinsic factors produced by both placental and decidual cells [14]. For example, human chorionic gonadotropin, tumor necrosis factor alpha (TNF-α), and adiponectin facilitate EVT migration by enhancing endometrial MMP-2,-9 activities and by inhibiting endometrial TIMP-1 secretion [12, 15, 16]. Conversely, the antifungal antibiotic trichostatin A limits trophoblast invasion by increasing the production of TIMP-1,-3 by ESCs and reducing the activity of endometrial MMP-2,-9 [11]. Hence, the local MMP/TIMP balance at the invasion site requires an appropriate interaction between trophoblasts and ESCs. Overall, the mechanisms underlying decidualization and decidual ECM remodeling are complex and have not yet been clearly elucidated.

Here, we focused on preimplantation factor (PIF) and its role in a successful, viable pregnancy. This factor is a small peptide secreted only by viable embryos and is present in the maternal circulation until term [17]. PIF is also expressed by the placenta [18]. Mondjie et al. have recently reported that PIF expression in trophoblastic cells is prominent in the earliest stages of pregnancy and then declines at term [19]. PIF’s major effects are essential for the initiation and maintenance of pregnancy by (i) promoting the development of cultured embryos, (ii) acting as a rescue factor against toxic-serum-induced embryo demise [20], (iii) regulating systemic immunity to promote embryo tolerance while preserving the mother’s ability to fight pathogens/disease and negating natural killer cell-induced toxicity [21], (iv) decreasing trophoblastic apoptosis through the p53 signaling pathway [22] and, (v) enhancing trophoblastic invasion [19]. Concerning this last point, it has been described that the pro-invasive effects of PIF in extravillous trophoblasts seemed to be mediated through different signaling pathways such as the mitogen-activated protein kinase (MAPK), phosphoinositide-3-kinase (PI3K), Janus-kinase signal transducer and transcription (JAK-STAT) signaling pathways [19]. Furthermore, two studies have shown that PIF can promote endometrial receptivity by regulating genes whose products are involved in inflammation, adhesion, and apoptosis, and by promoting the secretion of immune regulatory ligands and the phosphorylation of certain kinases as MAPK that actively condition the uterine environment [23, 24]. Taken as a whole, these data strongly suggest that PIF has a crucial role in embryo implantation. However, the molecular mechanisms underlying PIF’s effects on decidualization of human ESCs have not yet been fully characterized.

In this context, the primary objective of the current study was to specify PIF’s effect on human endometrium during the implantation window, in order to better understand the embryo–maternal dialog established during the early stages of pregnancy. To this end, we studied the in vitro effects of a synthetic analog of PIF (sPIF) at 50 nM on the human ESC decidualization program and the endometrial control of trophoblast invasion. This concentration was chosen since PIF is present at 50–150 ng/ml (30–100 nM) concentrations in the circulation of pregnant women [25] and sPIF is effective at modulating several decidual cell functions in the same concentration range [26–28].

## Materials and methods

### Materials

Dulbecco’s Modified Eagle’s Medium and Ham F-12 Nutrient Mix (DMEM/F12), Roswell Park Memorial Institute (RPMI) medium, progesterone, E2, penicillin, streptomycin, DNase type I, and bovine serum albumin were purchased from Sigma Chemical Co (Saint Louis, MO, USA). Fetal calf serum (FCS) was purchased from Invitrogen (Carlsbad, CA, USA). The sPIF (purity: 95%, as documented by HPLC and mass spectrometry) was produced by Biosynthesis (Lewisville, TX, USA). Superscript III RNase H-RT and primers were from Invitrogen, and RNase inhibitor was obtained from AMRESCO (Solon, OH, USA). The Nucleospin RNA II kit was obtained from Machery-Nagel (Düren, Germany). Trypsin was provided by Difco Laboratories (Detroit, MI, USA). Matrigel® was obtained from BD Biosciences (Le Pont-de-Claix, France) and collagenase A was from Boehringer (Mannheim, Germany). The selective, irreversible PI3K inhibitor wortmannin was purchased from Sigma Chemical Co. The suppliers of the various antibodies used here are described in the corresponding paragraphs below.

### Study population and tissue collection

A total of 34 normally cycling women (mean ± standard deviation age: 33.6 ± 0.5) undergoing biopsies for fertility evaluation were included in the study. Endometrial tissue was obtained by aspiration with a Pipelle (Pipelle de Cornier®, France) during the implantation window (day 20–25 of a spontaneous ovarian cycle). Women with anatomical abnormalities such as the presence of submucous fibroma or polyps, or a low endometrial thickness (< 7 mm during the luteal phase) were excluded. The inclusion criteria also included a good hormonal reserve and normal responses to ovarian stimulation. Indeed, all our study population has hormonal assays of FSH ≤ 8 mUI/ml, E2 ≤ 40 pg/ml, AMH ≥ 1.5 ng/ml, and an antral follicular count ≥12 on day 3 of the follicular phase. The study was approved by the local investigational review board (*Comité Consultatif de Protection des Personnes dans la Recherche Médicale, approval reference protocol 01–78*), Paris, France; reference: 01–78). All participants provided their written, informed consent before tissue sampling.

### Human ESC culture

Human ESCs were isolated and cultured as described by González and coworkers [29]. Briefly, tissue samples were minced into small pieces and digested in a two-step process. Tissues were incubated for 1 h at 37 °C in a phenol-red-free DMEM/F12 medium containing collagenase (0.1%), DNase type I (0.005%), penicillin (10 μg/mL), and streptomycin (100 U/mL). The supernatant was filtered through a 100 μm nylon screen and then centrifuged at 200 *g* for 10 min. A second enzymatic digestion was performed on undigested tissue for 10 min at 37 °C in DMEM/F12 medium containing trypsin (0.25%), DNase type I (0.1%), EDTA (0.03%), penicillin (10 μg/mL), and streptomycin (100 U/mL). The digested tissue was filtered through a 40 μm nylon screen and then centrifuged at 200 *g* for 10 min. Cell pellets from both digestions were pooled and centrifuged at 200 *g* for 10 min.

Cells were cultured in DMEM/F12 medium supplemented with streptomycin (10 μg/mL), penicillin (100 U/mL), and FCS (10%) at 37 °C in a 5% CO_2_ and 95% air atmosphere. When the cells reached confluence, they were cultured in the presence or absence of sPIF (50 nM or 100 nM) in differentiation medium containing DMEM/F12 with E2 (10^− 8^ M), P4 (10^− 6^ M), charcoal-stripped FCS (2%), penicillin (10 μg/mL), and streptomycin (100 U/mL) for 2 weeks. The medium was changed every 2 days, as described previously [16].

### Invasion assay for HTR-8/SVneo cells co-cultured with ESC-conditioned culture medium

The invasiveness of trophoblastic cells in response to ESC-secreted molecules was assessed using the HTR-8/SVneo immortalized EVT cell line (kindly provided by Dr. Nadia Alfaidy (CEA Grenoble, France), in agreement with Dr. Charles H. Graham, Queen’s University, Ontario, Canada). HTR-8/SVneo cells were cultured in RPMI medium supplemented with HEPES 1 M (2%), penicillin (100 U/mL), streptomycin (100 μg/mL), and FCS (10%) until they reached confluence. Invasion assays were performed in 24-well plates containing Matrigel®-coated polycarbonate membrane (pore size: 8 μm) invasion chamber inserts (Greiner Bio-One SAS, Courtaboeuf, France), according to the modified protocol described by Tapia-Pizarro and coworkers. Next, the HTR-8/SVneo cells were suspended (5 × 10^4^ cells per well) in 250 μL of conditioned medium (CM) from 15-day-decidualized ESCs treated (or not) with sPIF (50 nM). In order to validate our experimental conditions, media containing only E2 (10^− 8^ M) and P4 (10^− 6^ M) in the absence or presence of two positive controls i.e. FCS (10%) or adiponectin (250 ng/ml) were used as controls. RPMI medium supplemented with FCS (10%) was added to the lower well as a chemoattractant. After 48 h of incubation at 37 °C, medium containing non-invading cells was removed from the upper well. Invasive HTR-8/SVneo cells at the lower surface of the insert were washed and fixed with paraformaldehyde (4%) for 30 min. The nuclei were counterstained with 1 μg/mL Hoechst reagent and visualized with an inverted laser scanning confocal microscope (Leica white light laser TCS SP8-X, Leica Microsystems, Wetzlar, Germany). On each insert, invasive cells were counted on five randomly selected fields by using the post-imaging procedure in ImageJ software (National Institutes of Health, Bethesda, MD, USA). Invasive cells were defined as those whose nucleus exceeded 8 μm (i.e. the pore size).

### In vitro wound healing assay

ESCs were seeded at 1 × 10^5^ cells/cm^2^. After 15 days of differentiation (D15) in the presence or absence of sPIF (50 nM) in differentiation medium containing ovarian hormones (E2 at 10^− 8^ M and P4 at 10^− 6^ M), cell layers were wounded with a blade and washed three times with serum-free culture medium. The mark left by the blade on the plastic served as the migratory start line. Digital photographs of 6 different regions of each wound were taken before and 24 h after sPIF addition to serum-free culture medium. Cells that had migrated and repaired the wounded area were quantified using ImageJ software by measuring the width of the wound (mm/24 h) for each condition (in the presence of absence of sPIF).

### Zymography

After 15 days of culture with differentiation medium in the absence or presence of sPIF (50 nM) in differentiation medium containing ovarian hormones (E2 at 10^− 8^ M and P4 at 10^− 6^ M), total gelatinase activities from ESCs were analyzed by zymography. Aliquots of CM containing 60 μg of protein were resolved under non-reducing conditions in 10% polyacrylamide gels containing 1 mg/mL gelatin (Difco). Gels were washed in Triton X-100 (2.5%) for 30 min to remove SDS and incubated overnight at 37 °C in renaturing buffer (50 mM Tris–HCl, pH 7.5, 5 mM CaCl_2_, 150 mM NaCl, and 0.02% sodium azide). Gels were stained with Coomassie Brilliant Blue and destained in methanol/acetic acid (20%/5% v/v). Proteolytic activity was identified as a clear band on a blue background. The images were scanned, and the band intensities were quantified using ImageJ software.

### Reverse transcription - quantitative polymerase chain reaction (RT-qPCR)

ESCs were seeded into 12-well culture plates (3.5 × 10^5^ cells per well). After 3, 8 or 15 days of differentiation (D3, D8, D15) in the presence or absence of sPIF (50 nM) in differentiation medium containing ovarian hormones (E2 at 10^− 8^ M and P4 at 10^− 6^ M), total RNA (0.1 μg) from ESCs was extracted and reverse-transcribed, as described previously. RT-qPCR was performed using the C1000 Thermal Cycler (CFX96 real-time system; BioRad, Hercules, CA, USA) and the primer sets indicated in Table 1. The final concentration of primers was 25 μM. Three reference genes coding for ribosomal protein L13A (*RPL13A*), TATA box-binding protein (*TBP*) and β2-microglobulin were chosen as described previously [30]. For each sample, the concentration ratios (target/three reference mRNAs) were calculated using BioRad CFX Manager software (version 3.0) and expressed in arbitrary units, as described previously [16, 31].

**Table 1:** Primers used for RT-PCR

Primer set	Sequence	PCR product (bp)
**PROLACTIN**		
Sense	**5′** AGC CAG GTT CAT CCT GAA A **3′**	99
Antisense	**5′** TTC TCA GAG CGG AAA GAC GA **3′**	
**IGFBP-1**		
Sense	**5′** ATC ACA GCA GAC AGT GTG AGA **3′**	71
Antisense	**5′** CCA CGC AGA TGG GAA CCT TA **3′**	
**CONNEXIN-43**		
Sense	**5′** TTA AGC AAA AGA GTG GTG CCC **3’**	179
Antisense	**5′** GAC CCC TCT ACT CGT CAG AC **3’**	
**TIMP-1**		
Sense	**5′** GGG CTT CAC CAA GAC CTA CA **3’**	71
Antisense	**5**′ TGC AGG GGA TGG ATA AAC AG **3**’	
**TIMP-2**		
Sense	**5′** GAA GAG CCT GAA CCA CAG GT **3’**	85
Antisense	**5′** GGG GGA GGA GAT GTA GCA C **3’**	
**TBP**		
Sense	**5′** TGC ACA GGA GCC AAG AGT GAA **3’**	132
Antisense	**5′** CAC ATC ACA GCT CCC CAC CA **3’**	
**B2-MICROGLOBULIN**		
Sense	**5′** TGC TGT CTC CAT GTT TGA TGT ATC T **3’**	86
Antisense	**5′** TCT CTG CTC CCC ACC TCT AAG T **3’**	
**RPL13A**		
Sense	**5′** CCT GGA GGA GAA GAG GAA AGA GA **3’**	125
Antisense	**5′** TTG AGG ACC TCT GTG TAT TTG TCA A **3’**	

### Prolactin secretion

Prolactin secretion into the culture medium was measured using an automated immunochemiluminescence assay system (Alinity®, Abbott, Rungis, France). In order to compare the levels of prolactin secretion, the results were normalized per 1 μg of total protein. The protein concentration was measured according to Bradford’s method, with bovine serum albumin as the standard.

### Statistical analysis

Statistical analyses were performed on the raw data from 5 to 13 separate experiments. A non-parametric, paired Wilcoxon test was used to compare (i) control situations (in the absence of sPIF) at D3 and D15, and (ii) the effect of 50 nM sPIF vs. the control situation at a given time point.

## Results

### Effect of sPIF on human ESC decidualization

**Fig. 1:**
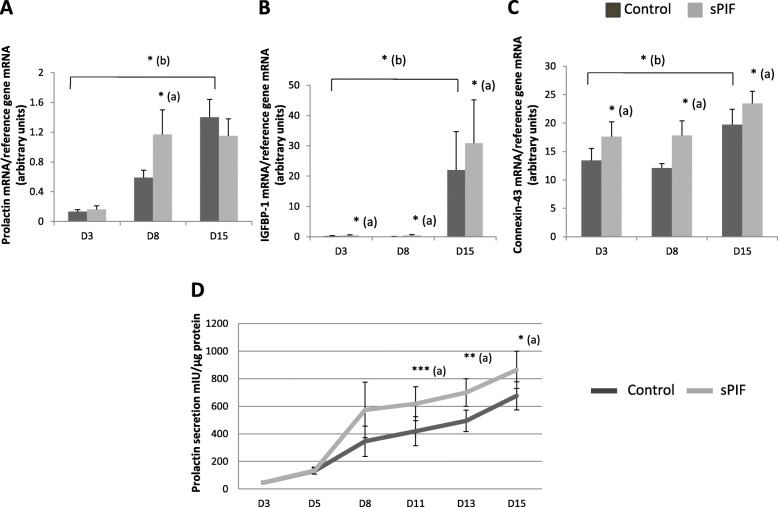
Effect of sPIF on human ESC decidualization. Human ESCs were cultured in DMEM/F12 medium supplemented with E2, P4 and (in some experiments) 50 nM sPIF for 15 days. **A**-**C** Total RNA was extracted after three (D3), eight (D8), and 15 days (D15) of cell decidualization. mRNA expression levels of *prolactin* (**A**), *IGFBP-1* (**B**), and *connexin-43* (**C**) were quantified by RT-qPCR, as described in the Materials and Methods section. The data are presented as the mean ± SEM of 6 to 10 separate experiments. **D** Prolactin secretion into the endometrial supernatants was measured after 3 days (D3), 8 days (D8), and 15 days (D15) of cell differentiation, as described in the Materials and Methods. The data are presented as the mean ± SEM of 8 to 13 separate experiments. The control values on D3, D5, D8, D11, D13, and D15 were 46.5 ± 8.7, 130.7 ± 22.5, 346 ± 110, 419 ± 105, 494.5 ± 77 and 676.4 ± 102 mIU/g protein, respectively. *: *P* < 0.05; **: *P* < 0.01; ***: *P* < 0.001 (Wilcoxon test); (a) Prolactin secretion in presence of sPIF vs. the control (lacking sPIF); (b) Prolactin secretion at D15 vs. D3 for control experimental condition (lacking sPIF)

*PRL, IGFBP-1,* and *CX-43* mRNA expression and prolactin secretion were used as biomarkers of sPIF’s effects on decidualization. After having validated our in vitro differentiation protocol (i.e. the observation of significant increases in the mRNA expression of *PRL*, *IGFBP-1* and *CX-43* during the ESC decidualization process; 11-, 100-, and 1.5-fold relative increases at D15, respectively) (Fig. 1A-C), we studied sPIF’s effect on this process. As shown in Fig. 1B-C, we found that *IGFBP-1* and *CX-43* mRNA expressions were significantly greater in the presence of sPIF at D3, D8 and D15. For *PRL* mRNA expression, this upregulatory effect was observed at D8 only (Fig. 1A). Furthermore, we observed significantly greater prolactin secretion in the presence of sPIF (50 nM) at D11, D13 and D15 (by a factor of 1.5, 1.4 and 1.3, respectively) (Fig. 1D). Similar results on prolactin secretion of ESCs were obtained with a higher sPIF concentration i.e. 100 nM (1.7 ± 0.4 fold-increase) (data not shown). So, we decided to use 50 nM of sPIF in the following experiments.

### PI3K pathway involved in sPIF’s pro-differentiation effect

**Fig. 2:**
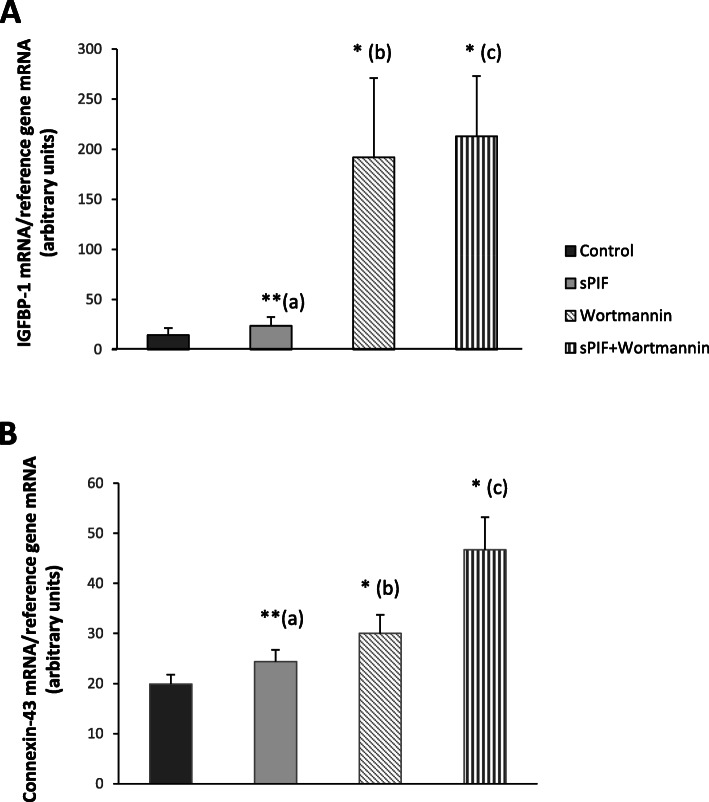
Effect of a PI3K inhibitor on sPIF-enhanced ESC decidualization. Human ESCs were cultured in DMEM/F12 medium supplemented with E2, P4, and the PI3K inhibitor wortmannin (10 μM) in the presence or in the absence of sPIF (50 nM) for 15 days. **A**-**B** Total RNA was extracted after 15 days (D15) of cell decidualization. mRNA expression levels of *IGFBP-1* (**A**), and *connexin-43* (**B**) were quantified by RT-qPCR, as described in the Materials and Methods section. The data are presented as the mean ± SEM of 4 to 11 separate experiments. *: *P* < 0.05; **: *P* < 0.005 (Wilcoxon test). (a) sPIF vs. control (lacking sPIF); (b) wortmannin vs. control (lacking sPIF); (c) sPIF vs. sPIF + wortmannin

To gain insight into the molecular mechanisms underlying the PIF-induced decidualization of human ESCs, we performed further experiments with a selective inhibitor of the PI3K signaling pathway. As show in Fig. 2, wortmannin exerted a significant effect per se on *IGFBP-1* and *CX-43* mRNA expression levels. Furthermore, we showed that sPIF’s pro-differentiation effect was not abrogated when the PI3K pathway was inhibited.

### Involvement of sPIF in endometrial motility

**Fig. 3:**
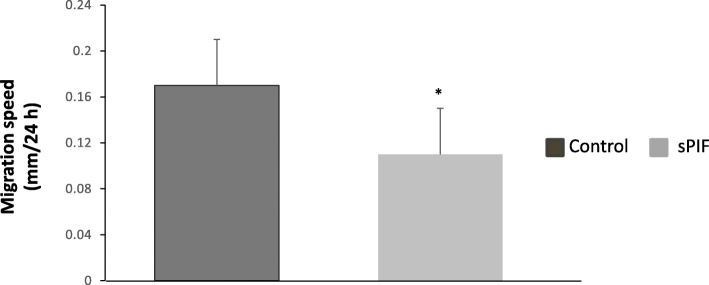
Effect of sPIF on human ESC motility. Human ESCs were cultured in DMEM/F12 medium supplemented with E2, P4 and (in some experiments) 50 nM sPIF for 15 days. Wound width was analyzed 0 and 24 h after wounding, as described in the Materials and Methods. The wound width data are expressed as the mean ± SEM of 5 separate experiments. *: *P* < 0.05 (Wilcoxon test) for sPIF vs. the control (lacking sPIF)

We used a wound repair assay to examine sPIF’s ability to modify the migratory properties of human ESCs. As shown in Fig. 3, cell motility into the denuded area under our experimental conditions was slightly decreased (by 18%) after exposure to sPIF for 15 days.

### Involvement of sPIF in the endometrial control of trophoblast invasion

**Fig. 4:**
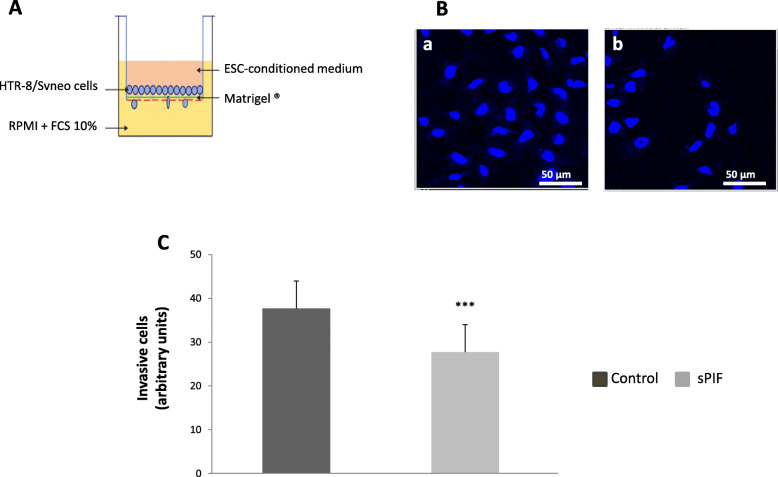
Involvement of sPIF in the endometrial control of trophoblast invasion by ESCs. PIF improves the endometrial control of trophoblastic migration. **A** Transwell migration assays of HTR-8/SVneo cells were performed as described in the Materials and Methods. **B** Representative microphotographs of 12 separate experiments showing fixed and DAPI stained HTR-8/SVneo cells on the bottom side of the transwell membrane. HTR-8/SVneo cells were cultivated with conditioned medium (CM) from decidualized ESCs exposed for 15 days in control condition (a) or with 50 nM sPIF (b). **C** HTR-8/SVneo cells were suspended in the presence of CM from decidualized ESCs having been exposed for 15 days in presence or absence of sPIF (50 nM) or in the presence of control medium, supplemented or not with FCS 10% or adiponectin (250 ng/ml). The data are presented as the mean ± SEM of 3–12 separate experiments. ***: *P* < 0.001 (Wilcoxon test) for sPIF vs. the control (lacking sPIF)

In order to determine how sPIF might be involved in the endometrial control of trophoblast invasion, we performed Matrigel® transwell invasion assays. We cultured the HTR-8/SVneo immortalized EVT cell line in the presence of CM conditioned by differentiating ESCs treated (or not) with sPIF (50 nM) for 15 days (Fig. 4A). As shown in Fig. 4B and C, we found that culture with CM from sPIF-treated ESCs was associated significantly lower invasive activity of HTR-8/SVneo cells (by 32%). In our experimental conditions, FCS (10%) and adiponectin (250 ng/ml) were used as positive control for EVT invasion.

### Effect of sPIF on endometrial MMP activities

**Fig. 5:**
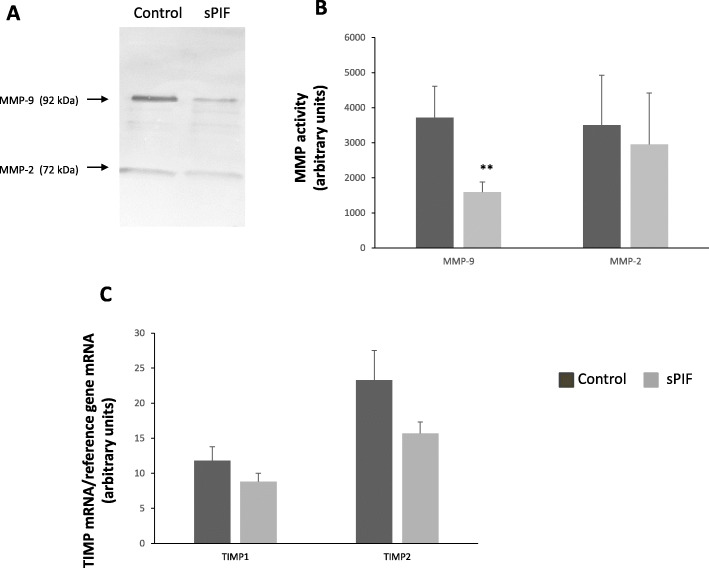
Effect of sPIF on endometrial TIMP mRNA expression and MMP activities. Human ESCs were cultured in DMEM/F12 medium supplemented with E2, P4 and (in some experiments) 50 nM sPIF for 15 days. **A** Activities of gelatinases (MMP-9 and MMP-2) in CM from decidualized ESCs having been exposed for 15 days in presence or absence of sPIF (50 nM), as described in the Materials and Methods. This figure shows one representative of 11 separate experiments. **B** Quantification of gelatin zymography results for MMP-2 and MMP-9 activities. The data are presented as the mean ± SEM of 5 to 8 separate experiments. **C** Total RNA was extracted after 15 days of cell differentiation. mRNA expression levels of *TIMP-1* and *TIMP-2* were quantified by RT-qPCR, as described in the Materials and Methods. The data are presented as the mean ± SEM of 11 separate experiments. **: *P* < 0.005 (Wilcoxon test) for sPIF vs. the control (lacking sPIF)

We next sought to specify the molecular mechanisms involved in sPIF’s anti-invasive action. Zymography assays revealed two bands, corresponding to the gelatinase activities of MMP-2 (72 kDa) and MMP-9 (92 kDa) in CM collected on D15 of cell culture. As shown in Fig. 5A and B, treatment with sPIF was associated with significantly lower MMP-9 activity on D15 (− 57%). However, under our experimental conditions, we did not observe any difference in MMP-2 activity in response to sPIF.

### Effect of sPIF on mRNA expression of endometrial TIMPs

Lastly, we focused on the mRNA expression of the invasion inhibitors TIMP-1 and TIMP-2, both of which are strongly expressed in human endometrium. The RT-qPCR assay did not reveal any significant changes in *TIMP-1* and *TIMP-2* mRNA expression after 15 days of culture in the presence of sPIF (Fig. 5C).

## Discussion

Early establishment of maternal-fetal crosstalk is critical for the progression of pregnancy. Many maternal and fetal factors have important roles during implantation and beyond. In the present study, we investigated the role of PIF - a small peptide exclusively secreted by competent embryos - on the decidualization process and on the endometrial control of trophoblast invasion.

We assessed ESC differentiation by measuring the mRNA expression of decidualization markers such as prolactin, IGFBP-1, and connexin-43. We observed significant upregulation of the expression of all three markers between D3 and D15 in response to the differentiation medium - indicating that ESCs are fully differentiated at D15. These results are in line with the literature data on in vitro ESC differentiation in the presence of E2 and P4 [32, 33]. We next studied the effect of sPIF on ESC decidualization. Our present results clearly show that sPIF increases biochemical ESC decidualization as evidenced by the significant increase in prolactin secretion by ESCs treated with sPIF. This upregulatory effect appears to be related to an effect of PIF on *PRL* gene transcription. However, PIF’s transitory effect on *PRL* mRNA expression is also associated with a longer-lasting effect on prolactin protein - suggesting that PIF influences the stabilization of *PRL* mRNA. The lack of correspondence between RNA and protein levels is not surprising and has been already described in the literature [34, 35]. More specifically, two different studies including a previous study of our laboratory clearly evidenced the mismatch between the prolactin mRNA expression and hormone release [16, 36]. We also demonstrated that sPIF upregulates *IGFBP-1* and *CX-43* mRNA expression during ESC differentiation and thus accentuated the decidualization of human ESCs. Our results are in line with previous genomic and proteomic studies in which PIF promoted endometrial receptivity by downregulating a gene specifically implicated in human endometrial receptivity i.e. the gene coding for phosphodiesterase 4B. Indeed, this enzyme specifically hydrolyses cAMP, a critical promotor of decidualization [23].

Many signaling pathways are known to be involved in human ESC decidualization. Furthermore, we previously reported that PIF’s role in trophoblast cells was mediated (at least in part) by the PI3K signaling pathway [19]. Hence, to gain insights into the molecular mechanisms involved in PIF-induced decidualization, we investigated the PI3K signaling pathway. We first showed that the specific PI3K inhibitor wortmannin per se upregulated *IGFBP-1* and *CX-43* transcription. These results were not unexpected because they are in line with a previous study of human stromal cells in which inhibition of the PI3K pathway was decisive in endometrial differentiation [37]. Secondly, our results revealed that sPIF’s prodifferentiation effect seems not be mediated by the PI3K pathway. We are currently seeking to determine the possible involvement of another signal transducer (Janus-kinase signal transducer and activator of transcription) in PIF’s control of the decidualization process. We effectively previously demonstrated in our laboratory that PIF promoted human trophoblast invasion by stimulating different signaling pathways including JAK-STAT transduction [19]. In addition, two genome-wide transcriptomic change studies revealed that decidualization of ESCs is a multiphasic process involving distinct transcriptional programs including JAK-STAT transduction [38, 39]. Finally, it has been described that the transcription factor STAT3 seems to play a critical role on decidualization process [40, 41].

Our experiments also demonstrated that sPIF downregulated the mobility of decidualized cells. Hence, PIF exerts a paracrine effect on endometrial stromal cells by promoting their differentiation and inhibiting their migration. A similar action has already been described for another secreted protein (prokineticin 1) in human ESCs [42, 43]. Thus, the present study highlights the involvement of the embryonic peptide PIF in endometrial receptivity.

Decidualized stromal cells also control trophoblast invasion [14, 33]. We therefore decided to determine whether sPIF could change the ability of decidualized ESCs to modulate this process. Our results clearly demonstrated that exposure to medium conditioned by ESCs cultured for 2 weeks in the presence of sPIF significantly decreased trophoblast invasion. In order to gain more information on the molecular level, we next investigated sPIF’s effect on MMP activities and TIMP mRNA expressions in human ESCs. Our data demonstrated that sPIF is significantly and specifically associated with low MMP-9 activity in ESCs but not with a difference in TIMP expression. These findings suggest that PIF could regulate the production of endometrial factors already described as negative regulators of human trophoblast invasion (e.g. transforming growth factor beta (TGFβ) family members and interferon-γ) [44]. At present, it is difficult to say whether PIF directly controls the secretion of endometrial factors or whether it has an indirect effect.

It has been previously described that sPIF exerted a pro-receptive effect by promoting the expression of α2β3 integrin, a critical implantation marker, in endometrial epithelial cells [24]. In this paper, we demonstrated that sPIF played a pro-differentiative role in ESCs. All these results suggest that PIF could be considered as a key regulator of endometrial functions.

Interestingly, we demonstrated in this paper that sPIF indirectly decreases the invasive capacities of human trophoblasts through secreted endometrial factors. In contrast, we previously showed that sPIF directly increases the invasive capacities of human trophoblasts [19]. So, the same molecule i.e. sPIF seems to exert a dual control of human trophoblast invasive capacities by maintaining pro-invasive and anti-invasive signals in placenta and endometrium, respectively. This dual control by PIF in decidual and trophoblast cells might limit cell migration and thus avoid excessive trophoblastic invasion. It is effectively well established that dysregulation of placental invasion is associated with several diseases of pregnancy, such as preeclampsia and intrauterine growth restriction [45, 46]. Our laboratory has previously reported that placental PIF protein levels in pregnancies affected by preeclampsia or intrauterine growth restriction were significantly lower than in gestational-age-matched controls [22]. Taken as a whole, our results suggest that PIF’s specific effects on human placenta and endometrium influence the onset of these pathologies by specifically modulating the trophoblast’s invasive capacities.

## Conclusions

In conclusion, our data suggest that PIF has a critical role in the establishment of human embryo implantation by i) increasing decidualization of ESCs and ii) limiting trophoblastic invasion.

## Data Availability

Literature search results are available from the authors on reasonable request.

## Abbreviations

CM: Conditioned medium
CX: Connexin
DMEM/F12: Dulbecco’s modified Eagle’s medium and Ham F-12 nutrient mix
E2: 17β-estradiol
ECM: Extracellular matrix
ESC: Endometrial stromal cell
EVT: Extravillous trophoblast
FCS: Fetal calf serum
IGFBP-1: Insulin-like growth factor-binding protein 1
MAP kinase: Mitogen-activated protein kinase
MMP: Matrix metalloproteinase
P4: Progesterone
PI3K: Phosphatidylinositol 3 kinase
PIF: Preimplantation factor
PRL: Prolactin
RPMI: Roswell Park Memorial Institute
TGFβ: Transforming growth factor beta
TIMP: Tissue inhibitor of metalloproteinases
TNF-α: Tumor necrosis factor alpha
